# Supplementary material for: Therapy-Acquired Clonal Mutations in Thiopurine Drug-Response Genes Drive Majority of Early Relapses in Pediatric B-Cell Precursor Acute Lymphoblastic Leukemia
Source: Diagnostics (Basel). 2023 Feb 25;13(5):884. doi: 10.3390/diagnostics13050884 (PMC10001400; doi:10.3390/diagnostics13050884)

Case2-Case14 Paired Clonal Tree & Fish Plot

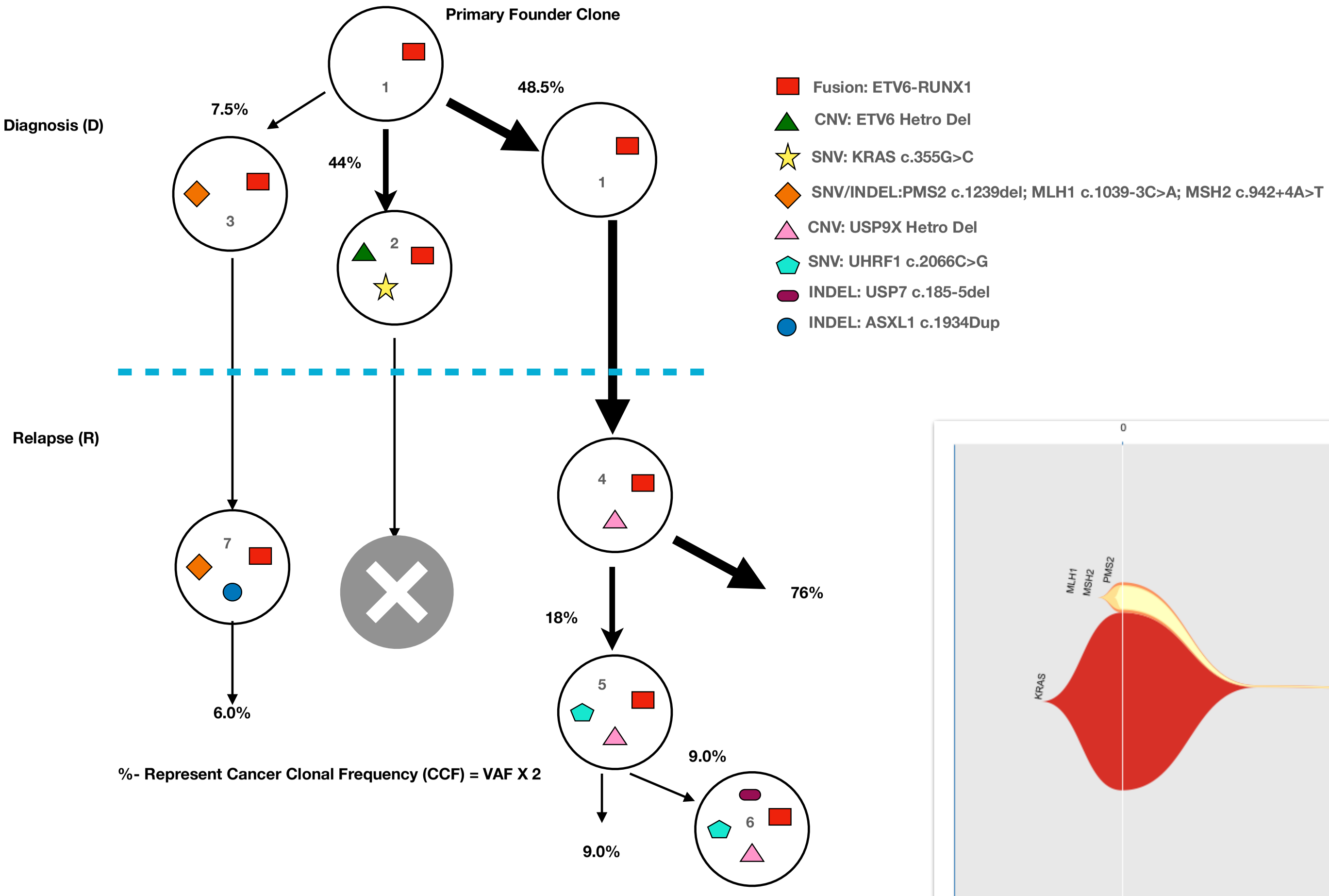

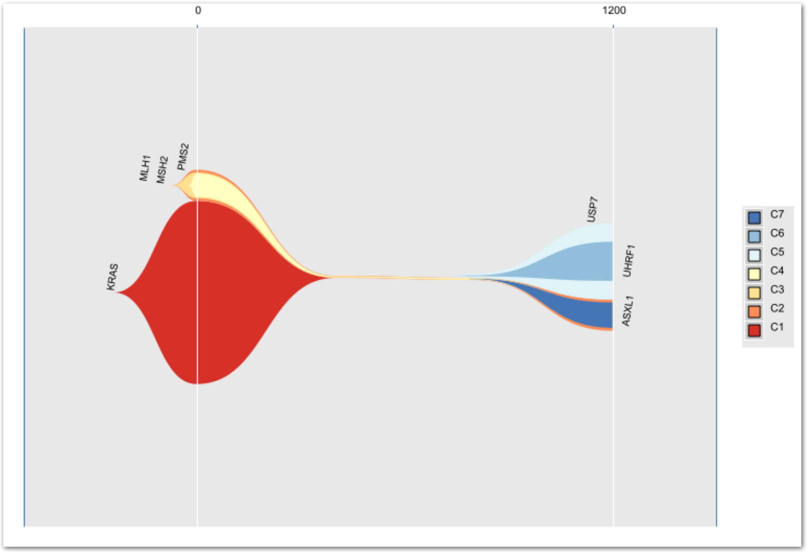

Case3-Case15 Paired Clonal Tree & Fish Plot

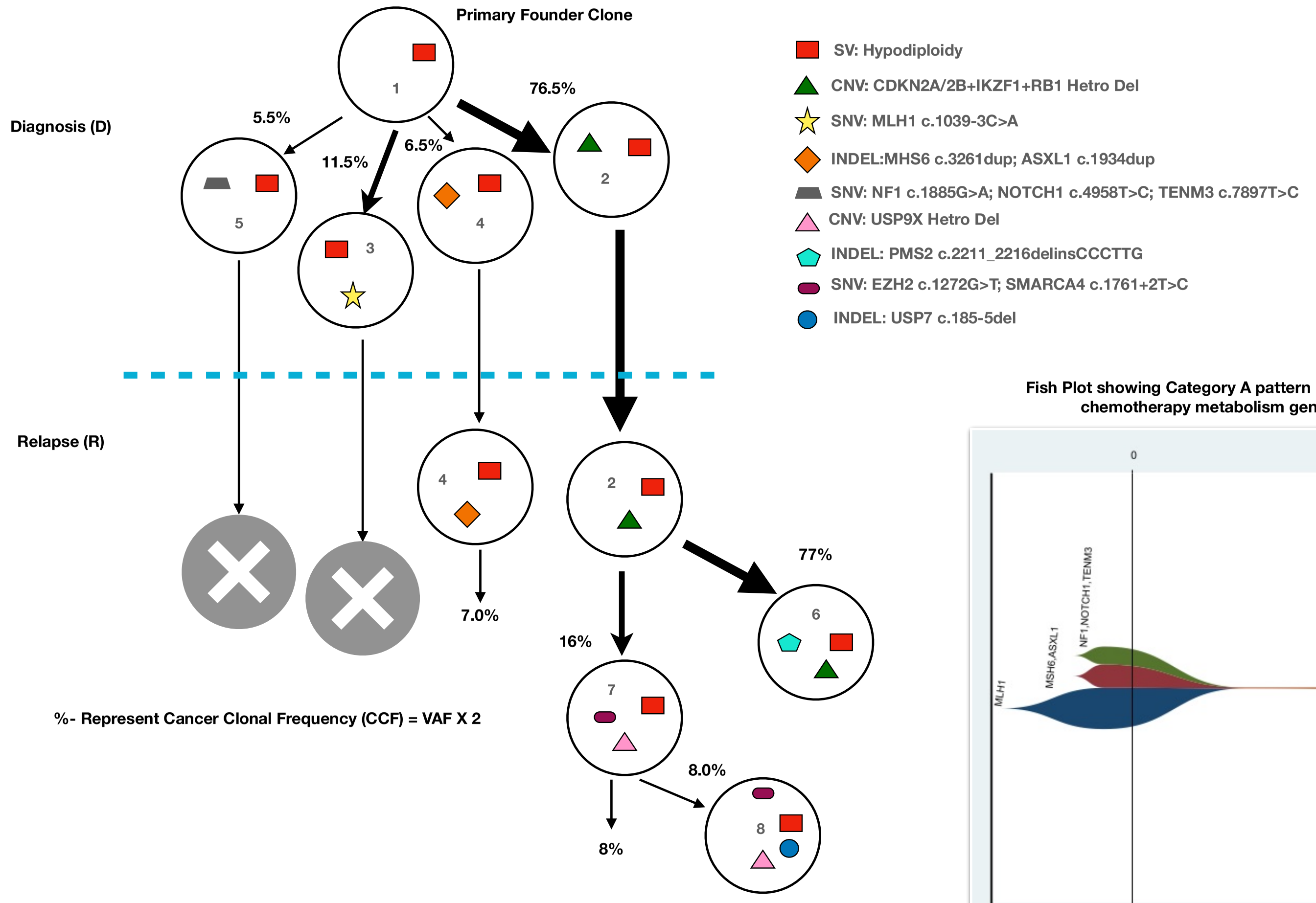

%- Represent Cancer Clonal Frequency (CCF) = VAF X 2

Fish Plot showing Category A pattern - Genetically distinct major clone at relapse in chemotherapy metabolism gene PMS2 under chemotherapy pressure

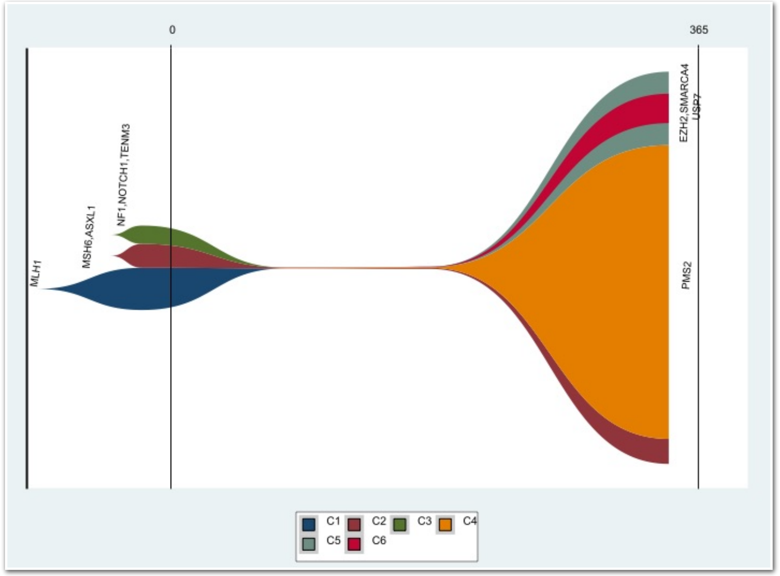

Case5-Case17 Paired Clonal Tree & Fish Plot

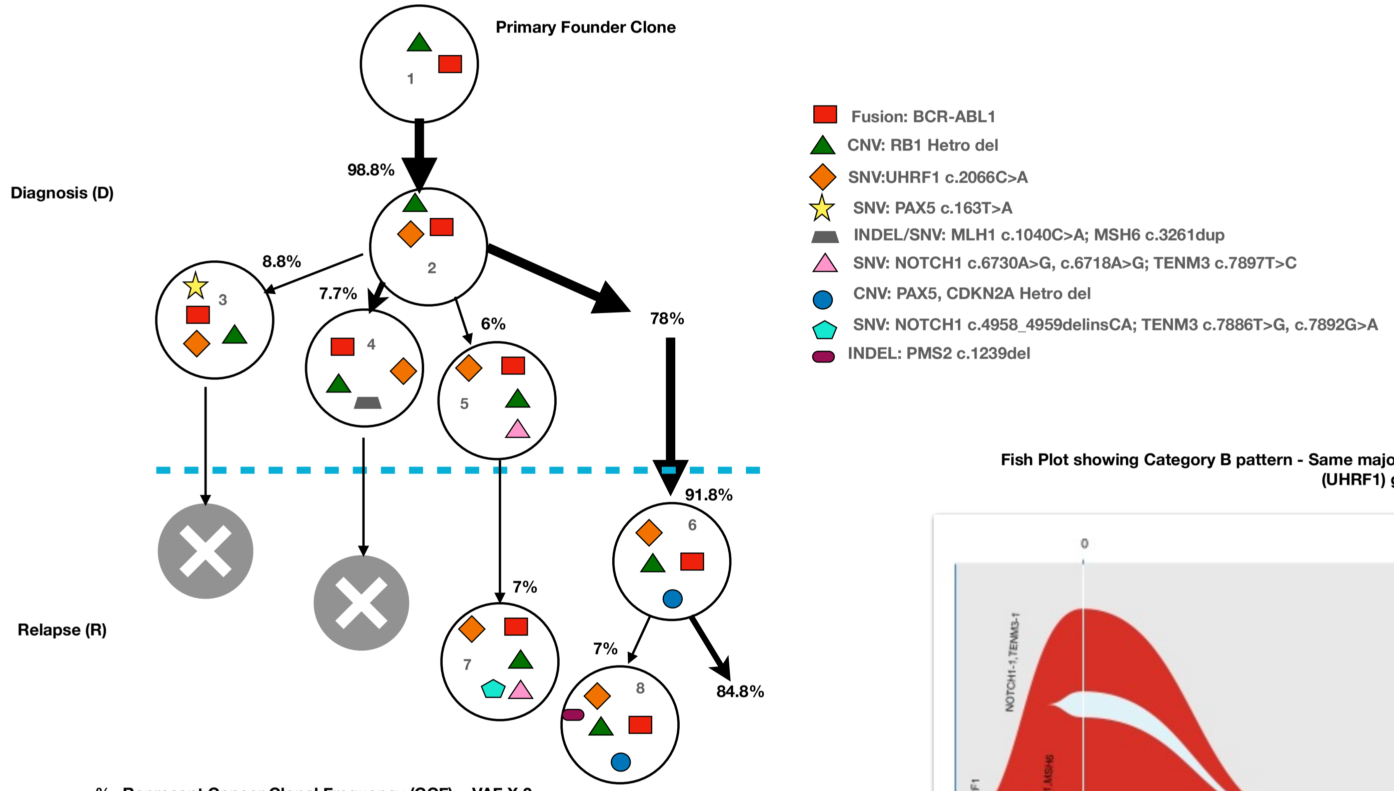

%- Represent Cancer Clonal Frequency (CCF) = VAF X 2

Fish Plot showing Category B pattern - Same major clone at diagnosis and relapse in epigenetic (UHRF1) gene

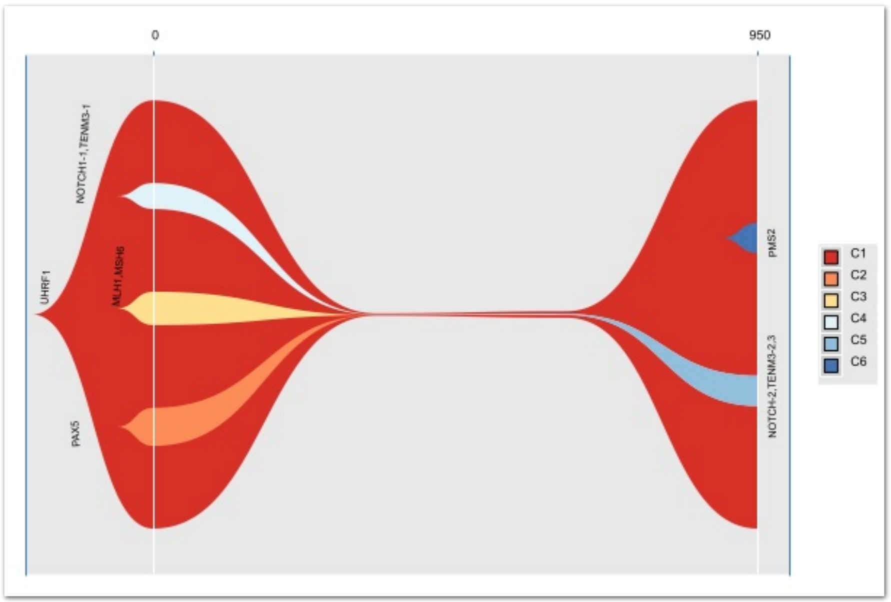

Case6-Case18 Paired Clonal Tree & Fish Plot

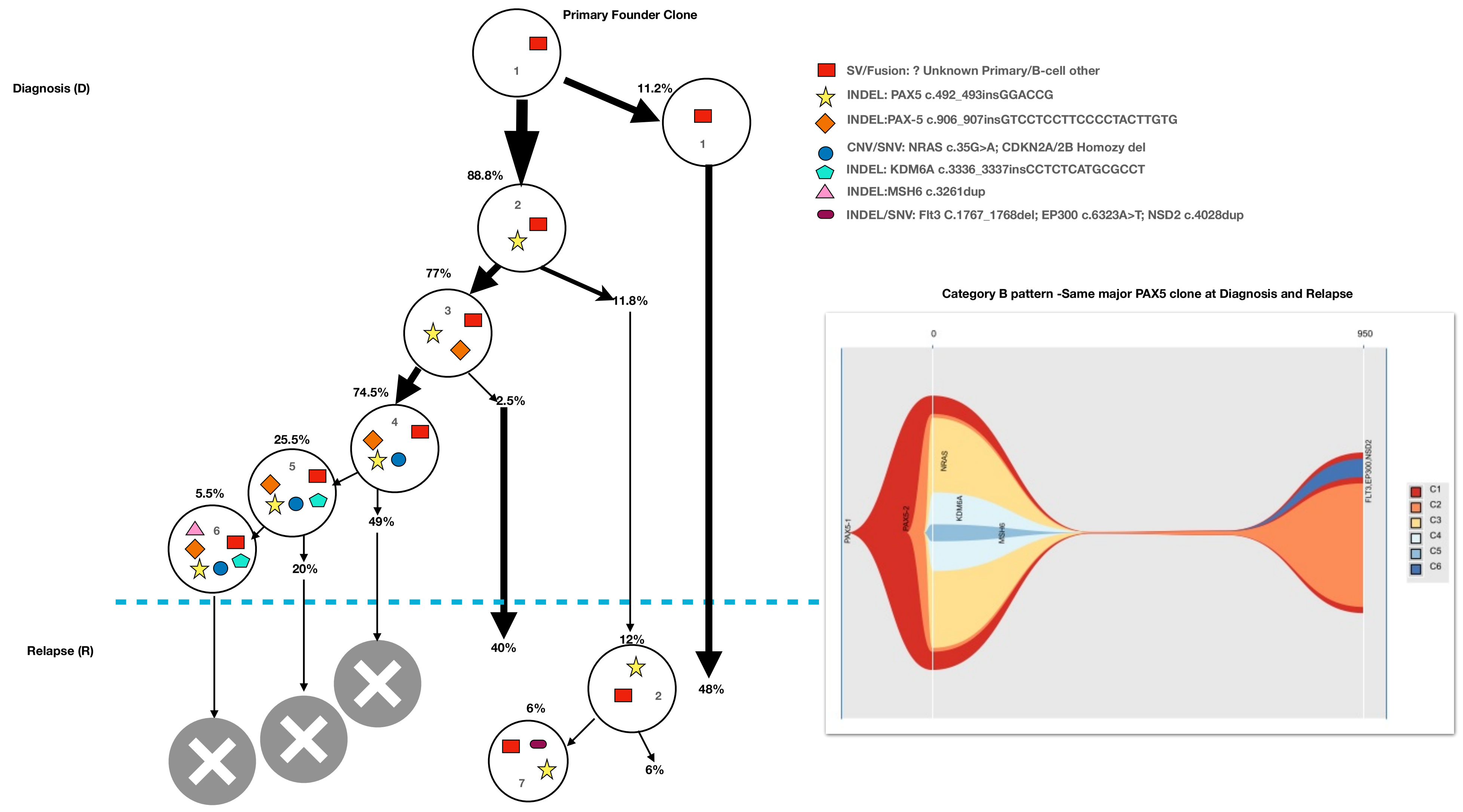

%- Represent Cancer Clonal Frequency (CCF) = VAF X 2

Case7-Case19 Paired Clonal Tree & Fish Plot

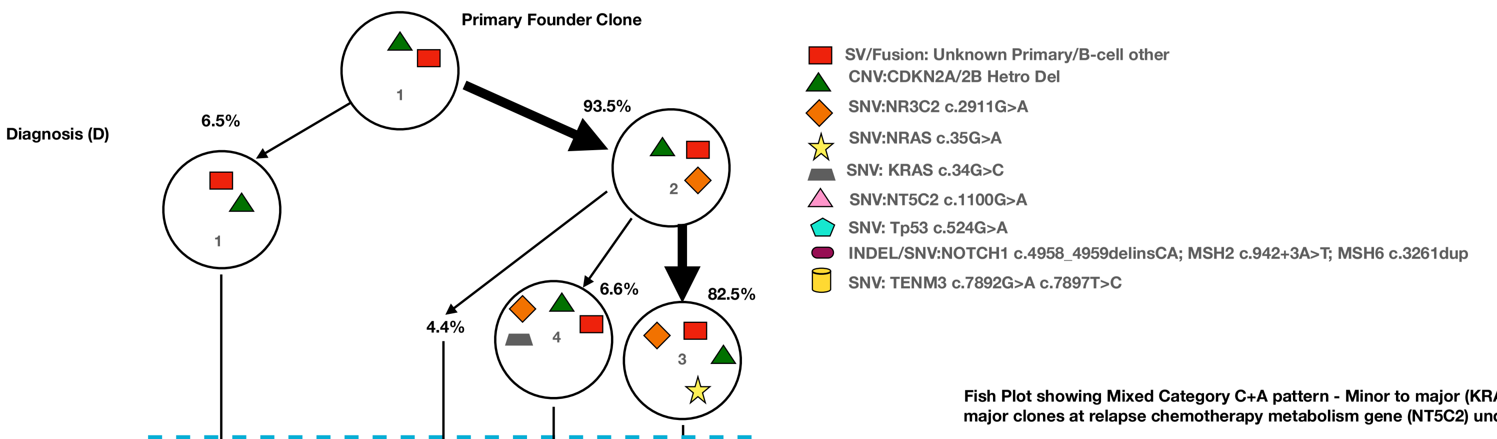

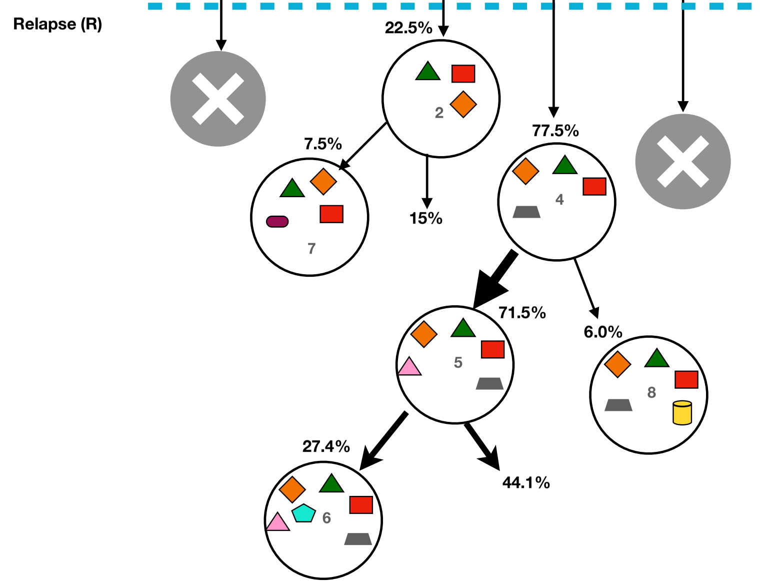

%- Represent Cancer Clonal Frequency (CCF) = VAF X 2

Fish Plot showing Mixed Category C+A pattern - Minor to major (KRAS) plus genetically distinct major clones at relapse chemotherapy metabolism gene (NT5C2) under chemotherapy pressure

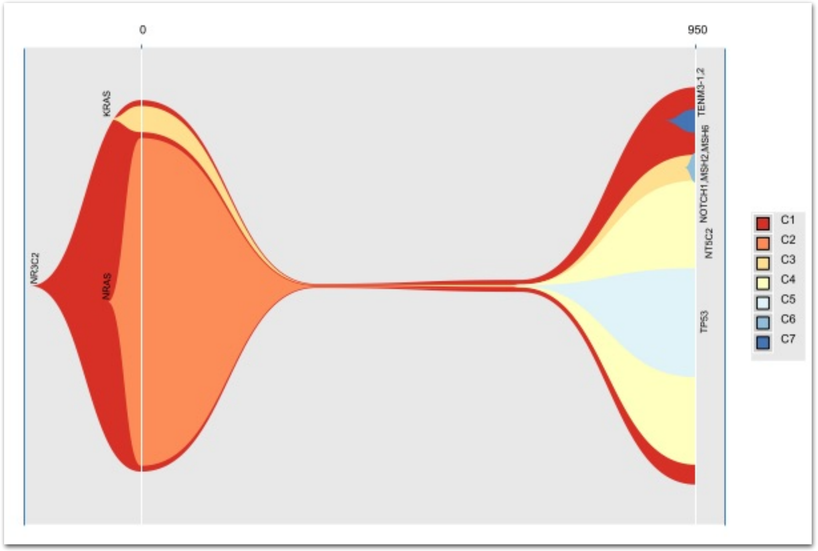

Case9-Case21 Paired Clonal Tree & Fish Plot

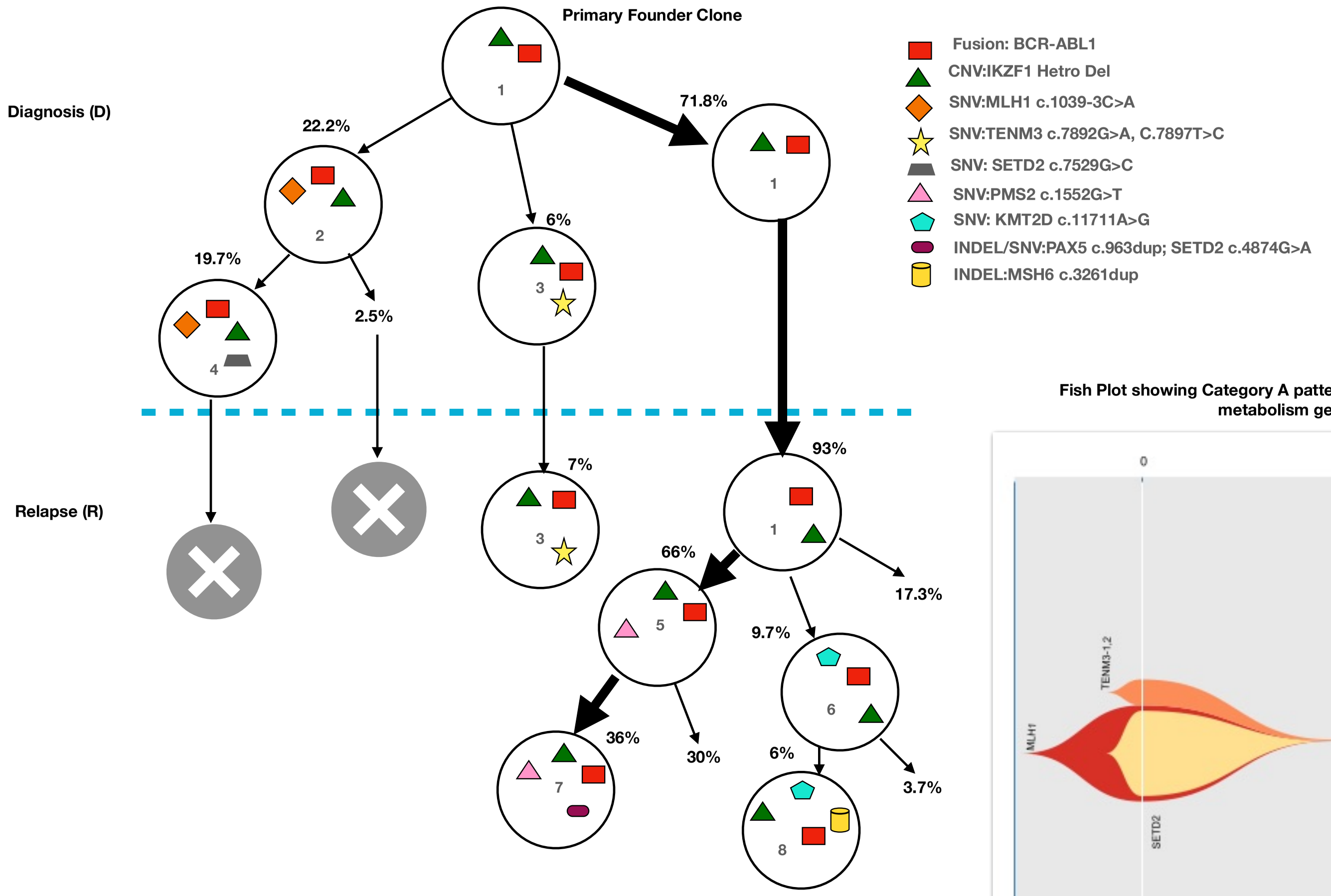

%- Represent Cancer Clonal Frequency (CCF) = VAF X 2

Fish Plot showing Category A pattern - Genetically distinct major clone at relapse chemotherapy metabolism gene (PMS2) under chemotherapy pressure

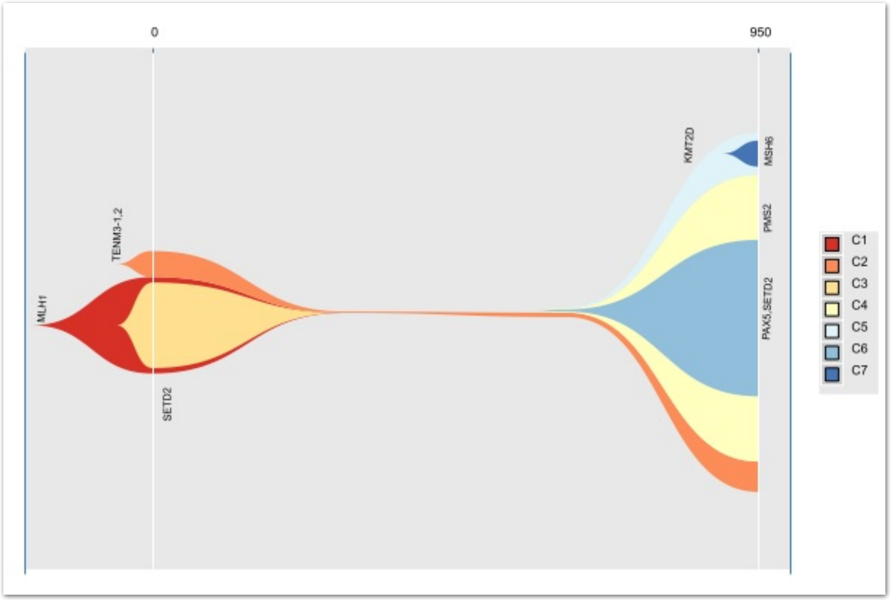

Case11-Case23 Paired Clonal Tree & Fish Plot

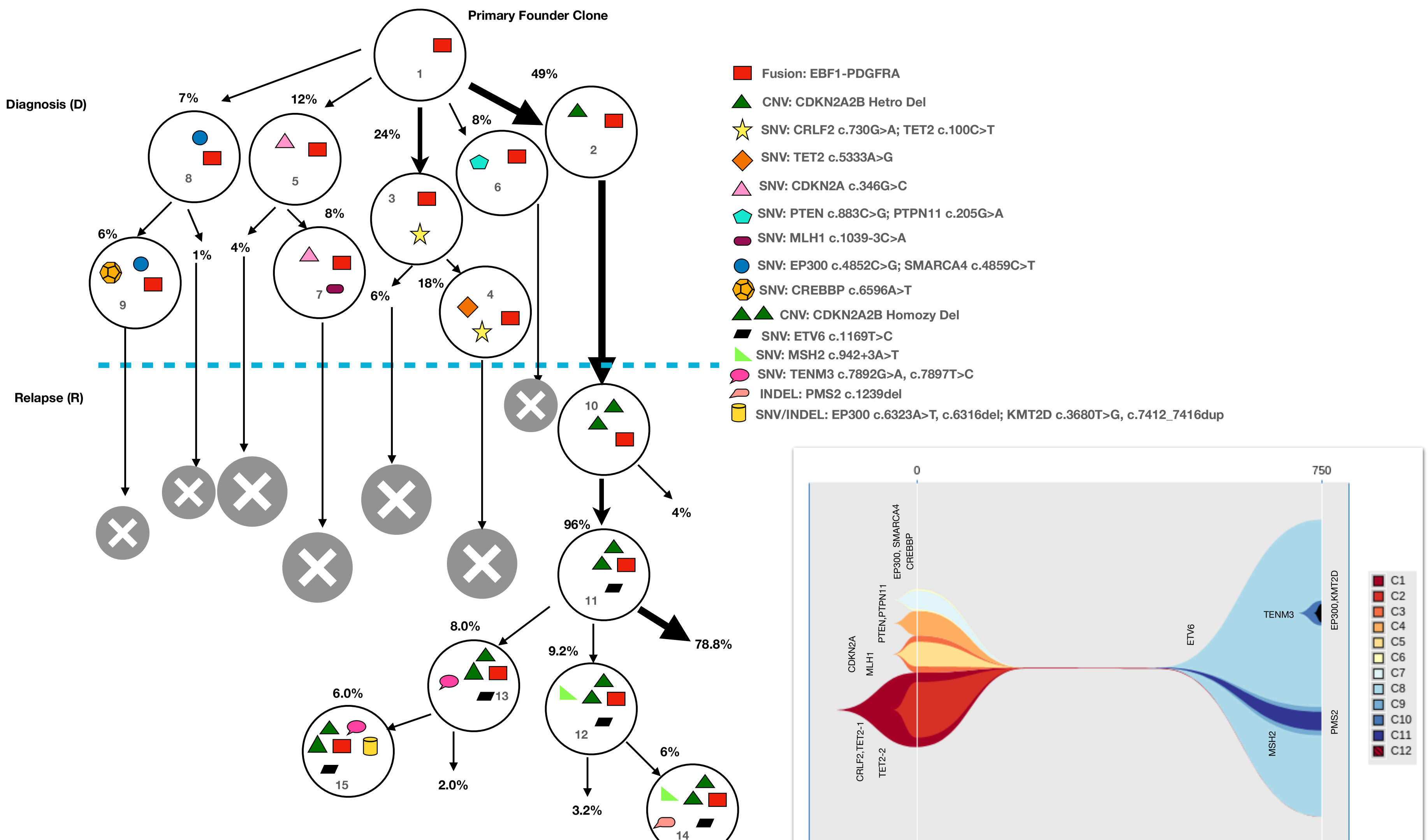

%- Represent Cancer Clonal Frequency (CCF) = VAF X 2

Case12-Case24 Paired Clonal Tree & Fish Plot

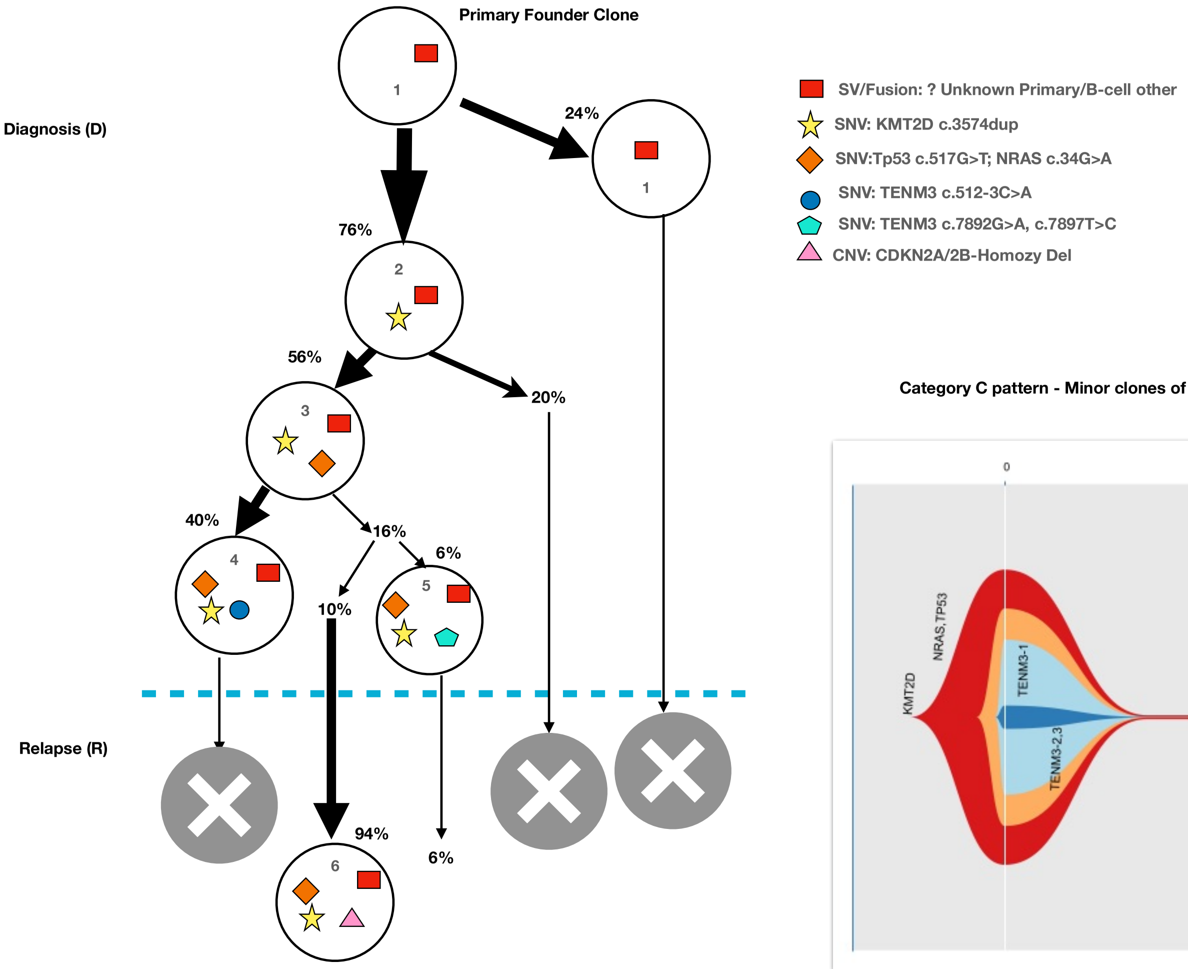

%- Represent Cancer Clonal Frequency (CCF) = VAF X 2

Category C pattern - Minor clones of NRAS and Tp53 becoming Major clones at relapse

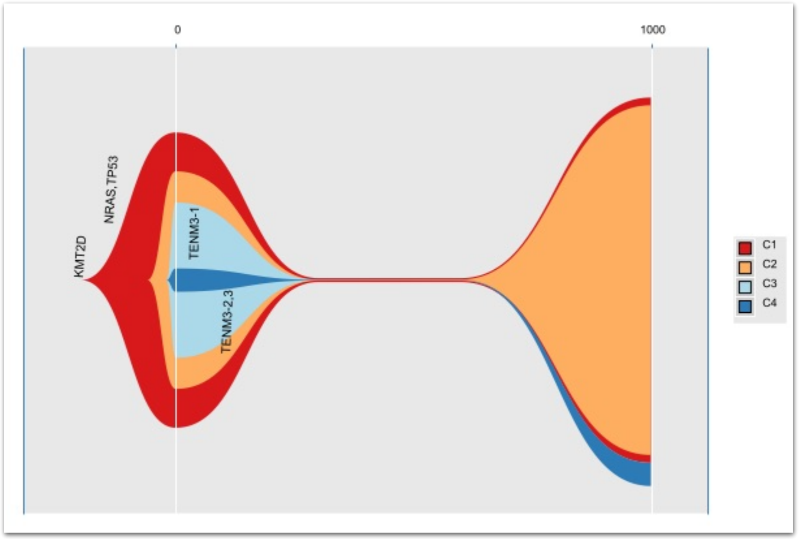

Case1-Case2-2nd run Paired Clonal Tree & Fish Plot

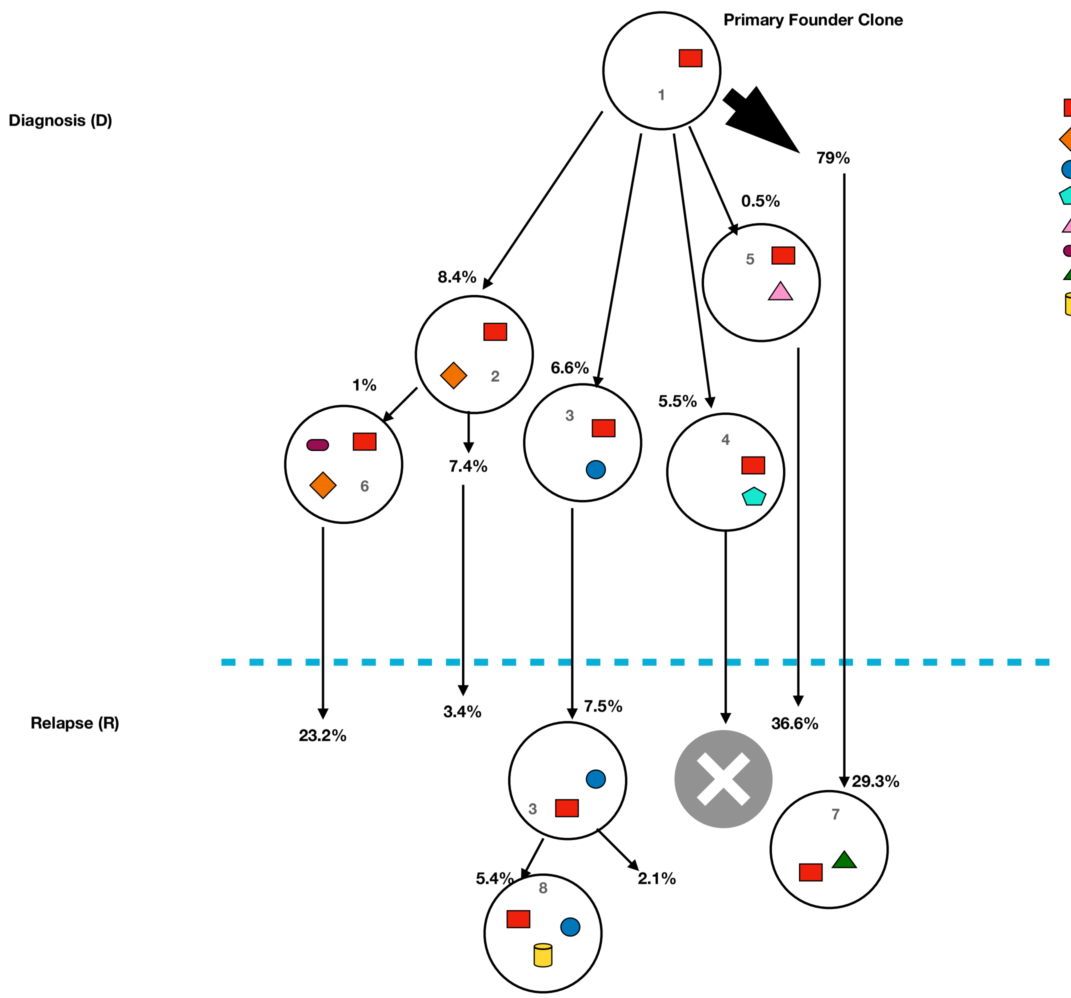

%- Represent Cancer Clonal Frequency (CCF) = VAF X 2

Fish Plot showing Category C pattern - Multiple minor clones at diagnosis becoming major or near major clones at relapse (RAS pathway-NRAS & KRAS)

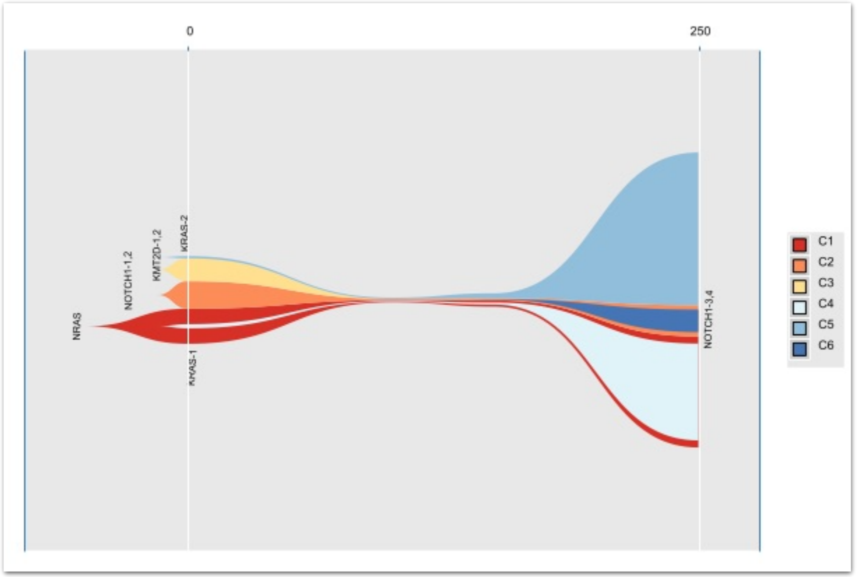

Case3-Case4-2nd run Paired Clonal Tree & Fish Plot

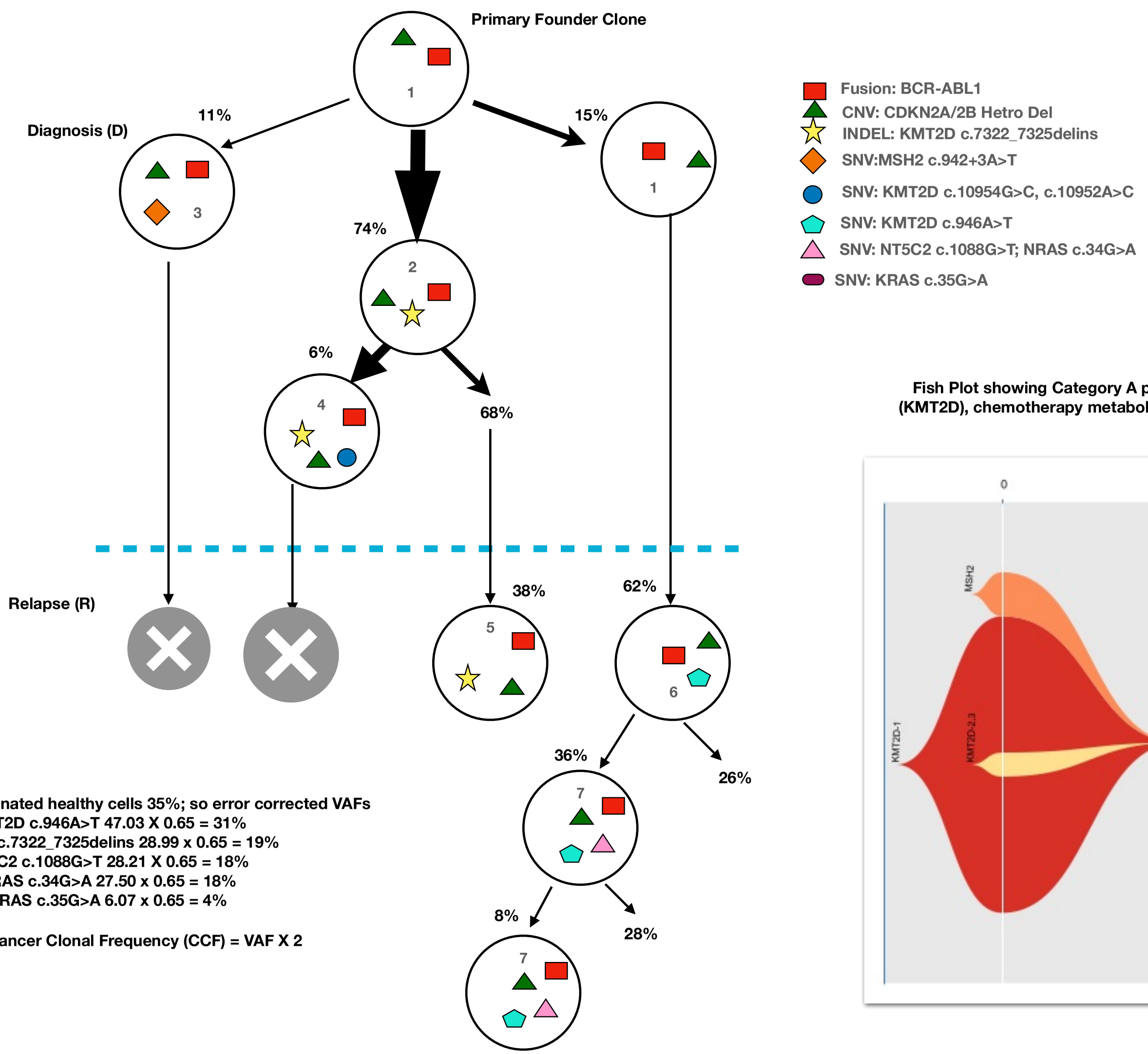

Relapse (R) Contaminated healthy cells 35%; so error corrected VAFs
KMT2D c.946A>T 47.03 X 0.65 = 31%
KMT2D c.7322_7325delins 28.99 x 0.65 = 19%
NT5C2 c.1088G>T 28.21 X 0.65 = 18%
NRAS c.34G>A 27.50 x 0.65 = 18%
KRAS c.35G>A 6.07 x 0.65 = 4%

%- Represent Cancer Clonal Frequency (CCF) = VAF X 2

Fish Plot showing Category A pattern - Genetically distinct major clones at relapse in epigenetic (KMT2D), chemotherapy metabolism gene (NT5C2) under chemotherapy pressure and RAS pathway

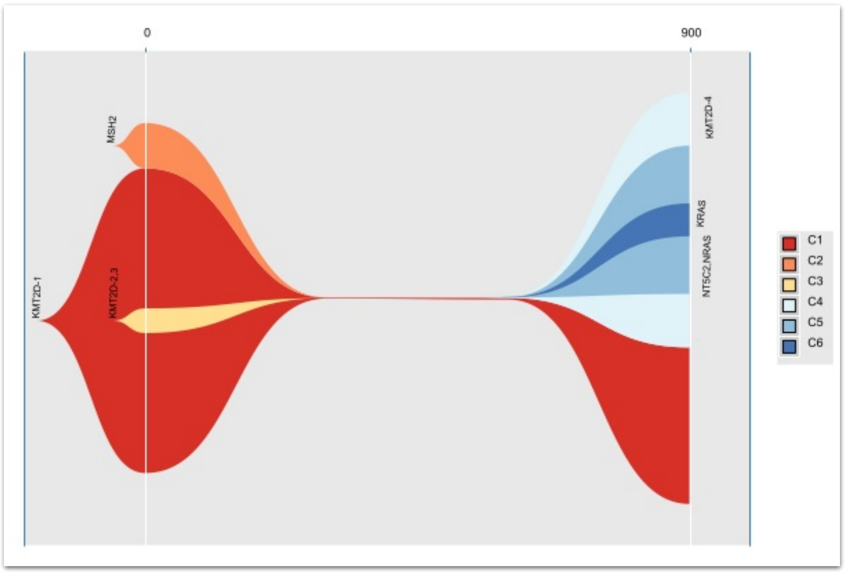

Case5-Case6 2nd run Paired Clonal Tree & Fish Plot

Diagnosis (D)

Diagnosis (D) Contaminated healthy cells 10%; so error corrected VAFs
MSH2 c.638T>A 52.22 X 0.90 = 47%
CREBBP c.1680del 50 x 0.90 = 45%
IKZF1 c.650C>T 49 x 0.90 = 44%
FPGS c.992C>G 49 x 0.90 = 44%
FPGS c.420C>G 41 X 0.90 = 37%
MSH2 c.942+3A>T 3.98 x 0.90 = 3.6%
NOTCH1 c.6730A>G,c.4958T>C 3.2 X 0.90 = 2.8%
TENM3 c.7892G>A,c.7897T>C 3 x 0.90 = 2.7%

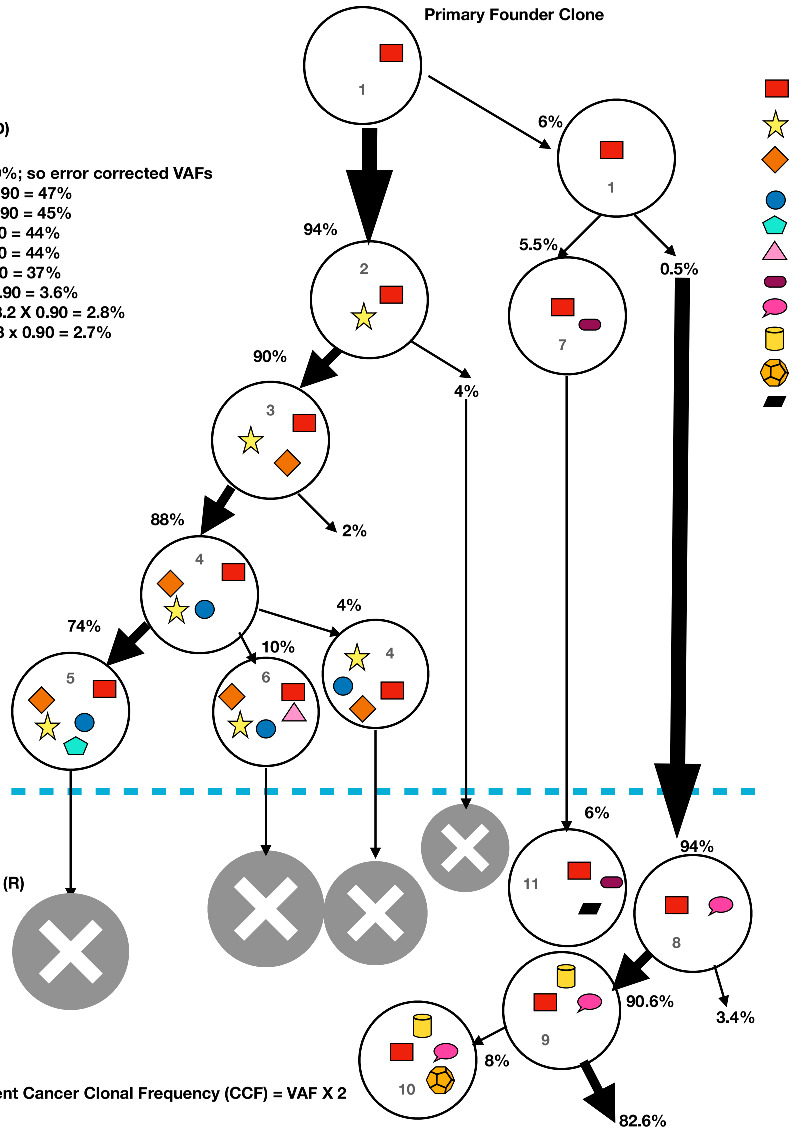

%- Represent Cancer Clonal Frequency (CCF) = VAF X 2

- Fusion: E2A-PBX (t1;19)
- SNV: MSH2 c.638T>A
- INDEL:CREBBPc.1680del
- SNV: IKZF1 c.650C>T; FPGS c.992C>G
- SNV: FPGS c.420C>G
- SNV: MSH2 c.942+3A>T
- SNV: TENM3 c.7892G>A, c.7897T>C; NOTCH1 c.6730A>G, c.4958T>C
- CNV: CDKN2B Homozy del
- SNV: KRAS c.34G>A
- SNV: EP300 c.3768T>G
- SNV: KMT2D c.13885A>C

Category A pattern - Genetically distinct clone (KRAS) at relapse due to chemotherapy pressure

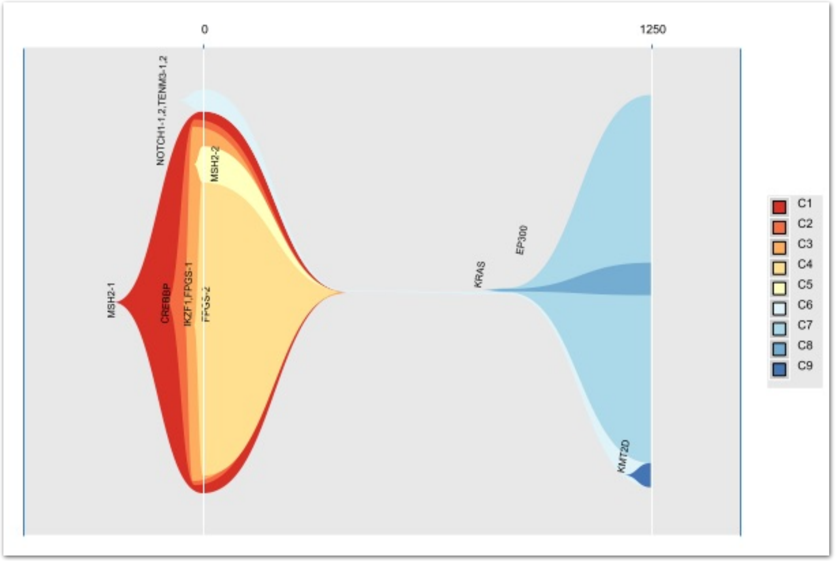

Case9-Case10-2nd run Paired Clonal Tree & Fish Plot

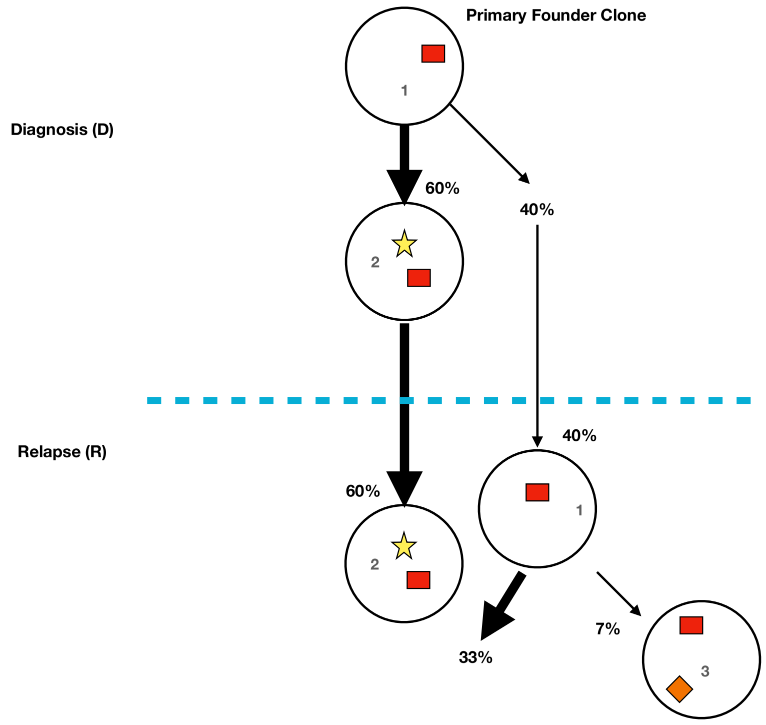

Fusion: TEL-AML1/t 12;21
SNV: KRAS c.436G>A; UHRF1 c. 1243G>A
INDEL/SNV:MSH2 c.942+3A>T; MSH6 c.3261dup

%- Represent Cancer Clonal Frequency (CCF) = VAF X 2

Fish Plot showing Category B pattern - Same major clones at diagnosis and relapse in RAS and epigenetic (UHRF1) pathway

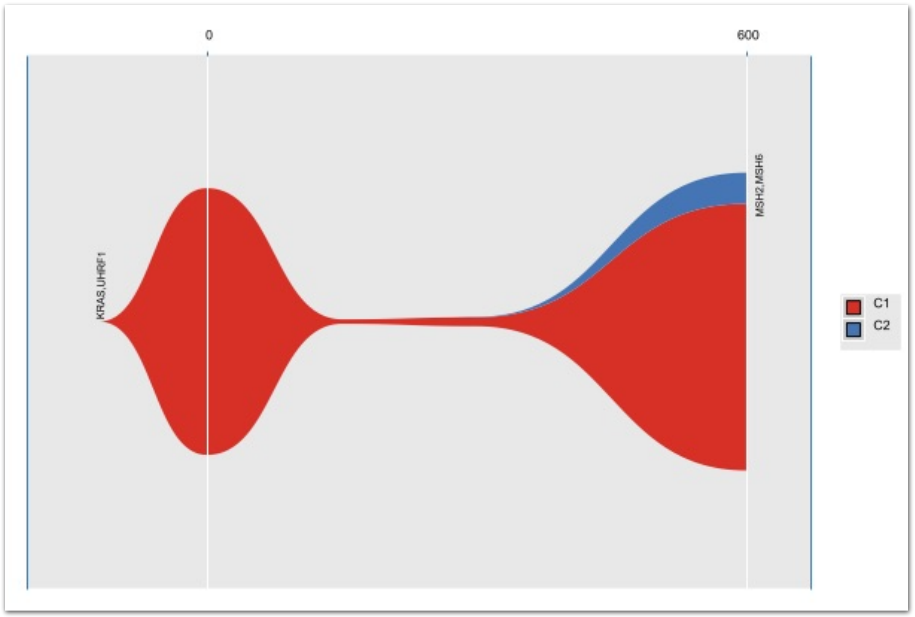

Case11-Case12-2nd run Paired Clonal Tree & Fish Plot

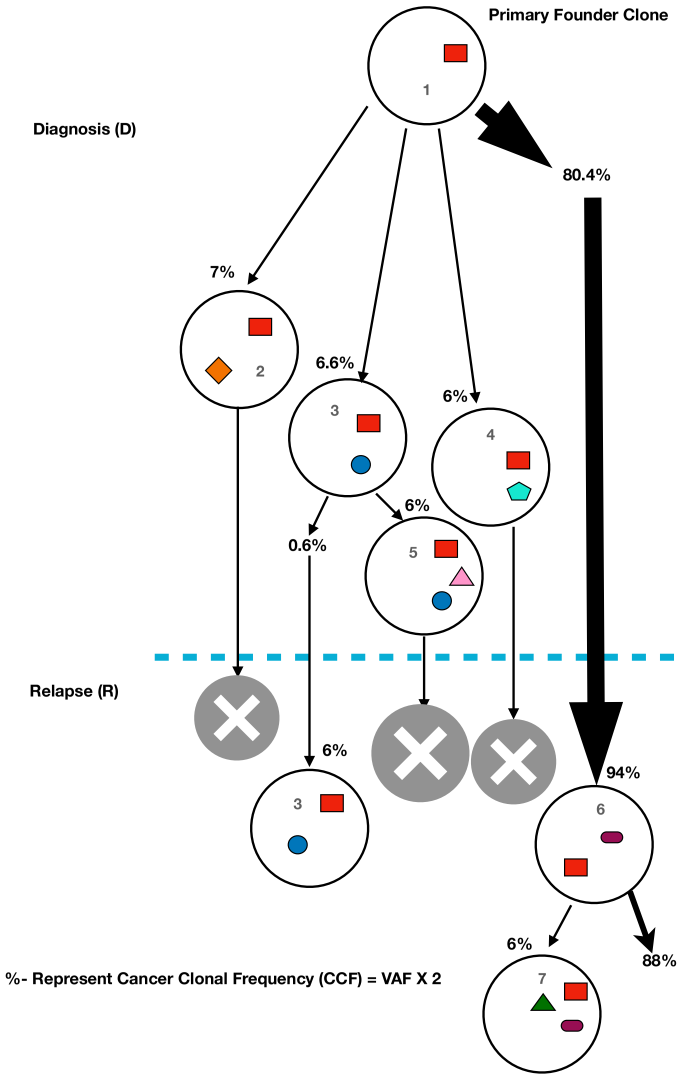

Fusion: ETV6-RUNX1
SNV:TENM3 c.7897T>C
INDEL/SNV: TENM3 c.7892G>A; NOTCH1 c.6718A>G c.4965_4966delinsGA
INDEL: NSD2 c.4028dup
INDEL NOTCH1 c.6723_6724delins
CNV/SNV: CDKN2A/2B Hetro del; ETV6 Homozy del; UHRF1 c.1781G>A
INDEL/SNV: EP300 c.1877G>A, c.6316del

Fish Plot showing Category A pattern - Genetically distinct major clones at relapse in epigenetic (UHRF1) pathway

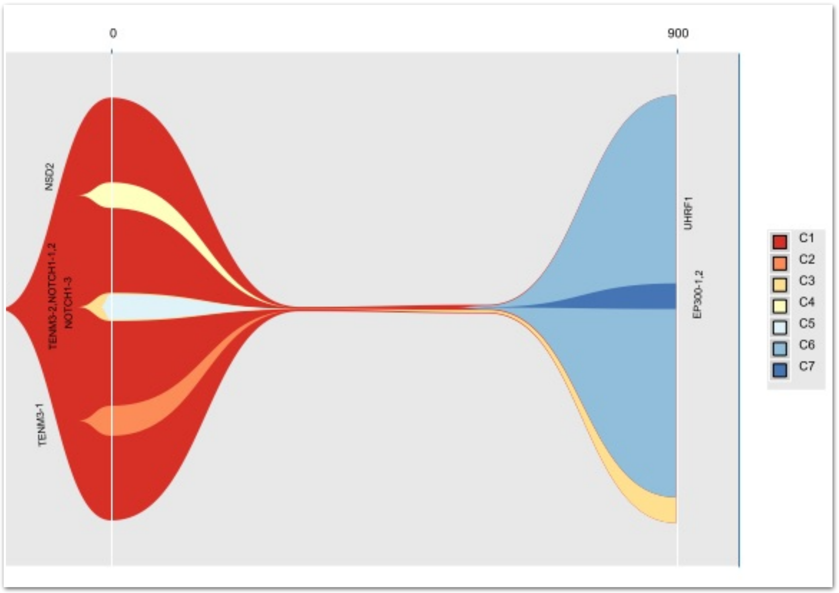

Case15-Case16-2nd run Paired Clonal Tree & Fish Plot

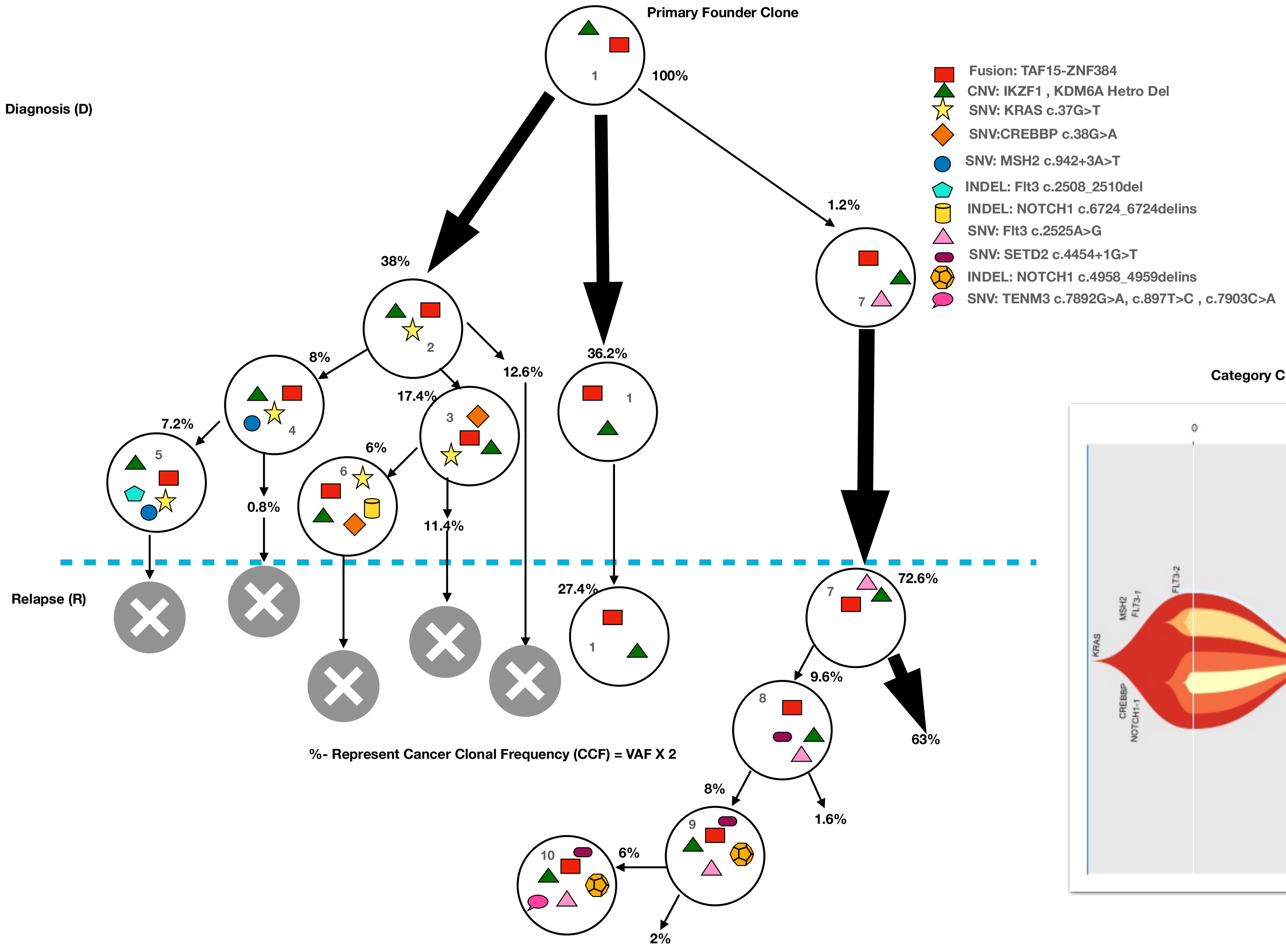

%- Represent Cancer Clonal Frequency (CCF) = VAF X 2

Category C pattern - Minor clone of Flt3 becoming Major clone at relapse

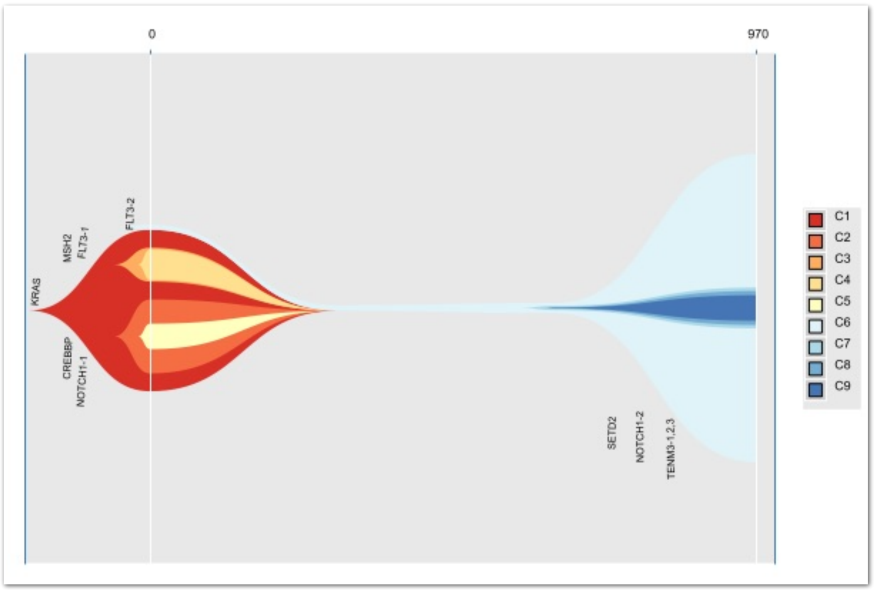

Supplement: Supplementary file 1 [file diagnostics-13-00884-s001.zip › Supplementary Figures.pdf]
