# Supplementary material for: Therapy-Acquired Clonal Mutations in Thiopurine Drug-Response Genes Drive Majority of Early Relapses in Pediatric B-Cell Precursor Acute Lymphoblastic Leukemia
Source: Diagnostics (Basel). 2023 Feb 25;13(5):884. doi: 10.3390/diagnostics13050884 (PMC10001400; doi:10.3390/diagnostics13050884)
Supplement: Supplementary file 1 [file diagnostics-13-00884-s001.zip › supplementary tables-new.pdf]

Supplementary Table S1: The GEP 15 gene panel along with housekeeping gene (18s) and PCR conditions to identify Ph-like signature using RQ-PCR

18S	*CRLF2*
CA6	***BMPR1B***
IFITM1	***S100Z***
CHN2	***PON2***
JCHAIN	***MUC4***
SPATS2L	***NRXN3***
TP53INP1	***ADGRF1***
[illegible]	[illegible]

STEP	TEMPERATURE	TIME	
UNG incubation	50 C	2 minutes	1
Enzyme activation	50 C	20 seconds	1
Denaturation	95 C	1 second	40
Anneal/ Extend	60 C	20 seconds	

Supplementary Table S2: Details of the targeted custom NGS RNA fusion panel for B-ALL (109 translocations/isoforms partners and 12 internal control genes)

FUSION	*PARTNERS*
BCR-ABL1	***Covered all breakpoints***
ABL1	***(partners- RANBP2, ETV6, FOXP1, NUP214, RCSD1, SFPQ, SNX2, ZMIZ1***
ABL2	***(partners- RCSD1, ZC3HAV1, ETV)***
KMT2A	***AFF1, MLLT1, MLLT3***
ZNF384	***EP300, TAF15,TCF3***
MEF2D	***HNRNPUL1, BCL9***
CSF1R	***MEF2D, SSBP2***
JAK2	***TERF2, STRN3, EBF1, BCR, ETV6, PAX5, PCM1, SSBP2***
PDGRFB	***ATF7IP, EBF1, ETV6***
PAX5	***AUTS2, FOXP1, ETV6), TCF3-HLH, ETV6-RUNX1, NTRK3-ETV6, IL2RB-MYH9***
TCF3-PBX1	***-***
PDGFRA-FIPL1	***-***
FGFR1-BCR	***-***

Supplementary Table S3: Details of genes/regions covered in the custom designed ArcherDx Next generation sequencing panel (Hot-spot regions- coloured brown; Entire coding regions- yellow & CNV covered- Y/yes)

S.No.	Gene	Transcript-ID (UCSC hg19 reference)	HOT-SPOT (Amino acid changes)/ Entire coding	CNV (Y/N)
1.	NRAS	NM_002524	p.G12A, p.G12C, p.G12D, p.G12S, p.G12V, p.G13D, p.G13V, p.Q61H, p.Q61K, p. Q61L	
2.	KRAS	NM_033360	p.A146T, p.A59E, p.G12A, p.G12D, p.G12R, p.G12S, p.G12V, p.G13D, p.I36M, p.K117N	
3.	PTPN11	NM_002834	p.A72T,p.A72V,p.D61G,p.E69K,p.E76K,p.G503R,p.S502A,p.T507K,p.T73I	
4.	NR3C2	-	ENTIRE CODING REGIONS	Y
5.	NT5C2	-	ENTIRE CODING REGIONS	
6.	NR3C1	-	ENTIRE CODING REGIONS	Y
7.	CREBBP	NM_004380	ENTIRE CODING REGIONS	Y
8.	WHSC1	NM_001042424	ENTIRE CODING REGIONS	Y
9.	PRPS1	-	ENTIRE CODING REGIONS	
10.	PRPS2	-	ENTIRE CODING REGIONS	
11.	MSH2	-	ENTIRE CODING REGIONS	
12.	PMS2	-	ENTIRE CODING REGIONS	
13.	FPGS	-	ENTIRE CODING REGIONS	Y
14.	CDKN2A	NM_001195132	p.D153fs,p.E10fs,p.M1K,p.R58_	Y
15.	CDKN2B	-	-	Y
16.	RB1	NM_000321	p.E398Q, p.S807_	Y
17.	TBL1XR1		-	Y
18.	ERG	-	-	Y
19.	EP300	-	ENTIRE CODING REGIONS	Y
20.	PHF6	NM_001015877	p.C305R, p.R274_	
21.	KMT2D	-	ENTIRE CODING REGIONS	
22.	KDM6A	-	ENTIRE CODING REGIONS	Y
23.	UHRF1	-	ENTIRE CODING REGIONS	Y

24.	SET2D	-	p.A2357fs, p.F1454fs, p.F1664Y, p.K2123fs, p.K2511fs, p.K2511Q, p.KKDSV1711fs, p.N719H, p.Q1734, p.S1531del, p.S1572_, p.S1838fs, p.T2101fs, p.Y1472_, p.Y2523fs	Y
25.	ATF7IP	-	-	Y
26.	SMARCA4	-	**ENTIRE CODING REGIONS**	
27.	ARID5B	-	**-**	Y
28.	USP7	-	Full coding regions	
29.	ASXL1	NM_015338	p.G642fs,p.L765fs,p.Q512_,p.W1037_	
30.	ABL1	NM_007313	p.E30D,p.E374G,p.E472G,p.N165S,p.T296N	Y
31.	Flt3	NM_004119	p.D593_F594delinsEC, p.D835A, p.D835Y, p.D839G, p.I836L, p.L576P, p.L576Q, p.V491L, p.V592D, p.Y591D, p.Y842C	
32.	EZH2	NM_004456	**ENTIRE CODING REGIONS**	
33.	ARID1A	-	-	Y
34.	NOTCH1	-	**ENTIRE CODING REGIONS**	
35.	PTEN	-	-	Y
36.	FBXW7		**ENTIRE CODING REGIONS**	
37.	STAG2	-	**-**	Y
38.	JAK2	NM_004972	p.E442Q, p.P503T, p.K585Q, p.N622I, p.D710E, p.I711V, p.S862C, p.R683G, p.R683S, p.T875N, p.P933Q, p.K1011	
39.	FGFR1	-	-	Y
40.	NF1	NM_001042492	p.G2334D, p.L626fs, p.T676fs, p.V1019fs, p.W2225_	
41.	RAG1	-	-	Y
42.	PAX5	-	p.E201fs, p.E205_, p.L58F, p.N29K, p.P80R, p.R140Q, p.V26fs, p.V26G, p.Y7C	Y
43.	IKZF1	NM_006060	p.E16fs,p.M459fs,p.R143Q,p.S17fs	Y
44.	ETV6	NM_001987	p.C338Y,p.H317fs,p.R359_,p.R55fs	Y
45.	LMO1		**ENTIRE CODING REGIONS**	
46.	ZEB2	NM_014795	p.H1038R	
47.	EBF1	-	-	Y
48.	LEF1	-	-	Y

49.	TAL1	-	-	Y
50.	TCF3	-	-	Y
51.	USH2A	NM_206933	p.D1788G, p.F1110V, p.P3590L,p.R3134Q,p.V49F	
52.	MLH1		**ENTIRE CODING REGIONS**	
53.	SUZ12	-	**-**	Y
54.	TET2		**ENTIRE CODING REGIONS**	
55.	PBX1	-	-	Y
56.	BTG1	NM_001731	p.103_103T>RP, p.E101fs	Y
57.	RUNX1	NM_001754	p.I337fs, p.L71fs, p.R166_, p.R201_, p.S114fs	Y
58.	DMD	NM_004006	p.C1267Y, p.M975L	Y
59.	ADD3	-	-	Y
60.	USP9X	NM_001039590	p.E1444fs, p.G1619fs	
61.	HTR3A	-	**ENTIRE CODING REGIONS**	
62.	TENM3	-	**ENTIRE CODING REGIONS**	
63.	MED12	-	**ENTIRE CODING REGIONS**	
64.	MLLT3	-	-	Y
65.	ARPP21	-	-	Y
66.	MTOR	NM_004958	p.A1459P, p.W1449R	
68.	CTNNB1			Y
69.	WNT9B	-	-	Y
70.	CRLF2	-	-	Y
71.	MLL2	NM_003482	**ENTIRE CODING REGIONS**	
72.	Tp53	NM_001126112	**ENTIRE CODING REGIONS**	Y
73.	MSH6	-	**ENTIRE CODING REGIONS**	Y
74.	VNN2		**ENTIRE CODING REGIONS**	

Supplementary Table S4: Somatic variant filter criteria used for data analysis

Filter	Parameter	Settings
1st Filter	AO (alternate observations)	>=5
	UAO (unique alternate observations)	>=3
	gnomAD AF (gnomeAD global population allele frequency)	<=0.05
	consequence	coding sequence variant, splice site
	AF (allele fraction)	>=0.027
2nd Filter (Manual)	Variants reported as artefacts, strand biases	Excluded
	Germline VAFs (49-50 or 90-100% VAFs in both diagnosis and relapse samples) as confirmed by remission sample DNA testing	Excluded
	Variants belonging to Tier IV (benign) or Tier III (VUS but evidence in favour of likely benign)	Excluded
